# The Dialogue of the Host-Parasite Relationship: *Leishmania* spp. and *Trypanosoma cruzi* Infection

**DOI:** 10.1155/2015/324915

**Published:** 2015-05-18

**Authors:** Carlos Gustavo Vieira de Morais, Ana Karina Castro Lima, Rodrigo Terra, Rosiane Freire dos Santos, Silvia Amaral Gonçalves Da-Silva, Patrícia Maria Lourenço Dutra

**Affiliations:** ^1^Laboratório de Bioquímica de Protozoários e Imunofisiologia do Exercício, Disciplina de Parasitologia, DMIP, FCM, Universidade do Estado do Rio de Janeiro, Avenida Professor Manuel de Abreu 444, Pavilhão Américo Piquet Carneiro, 5° andar, Vila Isabel, 20550-170 Rio de Janeiro, RJ, Brazil; ^2^Programa de Pós Graduação em Microbiologia/FCM/UERJ, Avenida Professor Manuel de Abreu 444, Pavilhão Américo Piquet Carneiro, 3° andar, Vila Isabel, 20550-170 Rio de Janeiro, RJ, Brazil; ^3^Programa de Pós Graduação em Fisiopatologia Clínica e Experimental/FCM/UERJ, Avenida Professor Manuel de Abreu 444, Pavilhão Américo Piquet Carneiro, 5° andar, Vila Isabel, 20550-170 Rio de Janeiro, RJ, Brazil; ^4^Laboratório de Imunofarmacologia Parasitária, Disciplina de Parasitologia, DMIP, FCM, Universidade do Estado do Rio de Janeiro, Avenida Professor Manuel de Abreu 444, Pavilhão Américo Piquet Carneiro, 5° andar, Vila Isabel, 20550-170 Rio de Janeiro, RJ, Brazil

## Abstract

The intracellular protozoa* Leishmania *spp. and* Trypanosoma cruzi* and the causative agents of Leishmaniasis and Chagas disease, respectively, belong to the Trypanosomatidae family. Together, these two neglected tropical diseases affect approximately 25 million people worldwide. Whether the host can control the infection or develops disease depends on the complex interaction between parasite and host. Parasite surface and secreted molecules are involved in triggering specific signaling pathways essential for parasite entry and intracellular survival. The recognition of the parasite antigens by host immune cells generates a specific immune response.* Leishmania *spp. and* T. cruzi *have a multifaceted repertoire of strategies to evade or subvert the immune system by interfering with a range of signal transduction pathways in host cells, which causes the inhibition of the protective response and contributes to their persistence in the host. The current therapeutic strategies in leishmaniasis and trypanosomiasis are very limited. Efficacy is variable, toxicity is high, and the emergence of resistance is increasingly common. In this review, we discuss the molecular basis of the host-parasite interaction of* Leishmania *and* Trypanosoma cruzi *infection and their mechanisms of subverting the immune response and how this knowledge can be used as a tool for the development of new drugs.

## 1. Host-Parasite Interaction 

Parasitic diseases are some of the greatest public health problems in developing countries. Several of these diseases are neglected, either because of their incidence in countries with little purchasing power or their low visibility. In general, the majority of these countries are located in the tropical zone. The climates of these areas contribute to the development of parasitic infections because humidity and high temperatures provide the necessary conditions for vector and protozoan growth [1].

All mammalian hosts are at risk of infection by viruses, bacteria, fungi, and parasites. The host-parasite relationship is the most important factor in determining whether an infection is successful or is resolved by the host. Several mechanisms are involved in this complex interaction, and aspects of both the host and the parasite are essential. Some parasites have evolved evasive mechanisms, such as intracellular infection, as in the case of the genus* Leishmania* and* Trypanosoma cruzi*, protozoa parasites belonging to family Trypanosomatid, order Kinetoplastida. These parasites are among the most important agents of neglected tropical diseases [2]. They are heteroxenic and infect two host types: vertebrates and invertebrates [3, 4]. Throughout their life cycle, they progress through several forms, including epimastigotes and metacyclic trypomastigotes, which are found inside the Triatominae vector of* T. cruzi* and procyclic and metacyclic promastigotes, which are found inside the Phlebotominae vector of* Leishmania* genus [3, 4]. Amastigotes are the intracellular form of the both parasites and are found inside the vertebrate host. Additionally,* T. cruzi* presents the blood trypomastigote forms in this host [3, 4].

*Leishmania* is responsible for a group of cutaneous and visceral infections known as leishmaniasis. These parasitoses are endemic in 98 countries distributed in Latin America, South and Central Asia and sub-Saharan Africa [5], where approximately 350 million people are threatened with contracting this infection. The annual incidence is estimated at 1.6 million, and the prevalence is 12 million [6].

*Trypanosoma cruzi* causes Chagas disease. An estimated 10 million people are infected by* T. cruzi*, mostly in Latin America, where Chagas disease is endemic, and more than 25 million people are danger of contracting this parasitosis [6].

The first step in the interaction between the host and these intracellular protozoa parasites is the binding of the parasite to the host cell. These protozoa have a variety of surface and secreted molecules used to attach and enter mammalian cells. Several of these molecules are involved in triggering specific signalling pathways essential for parasite entry and intracellular survival. Scientific advances in this area have identified factors critical to parasite virulence and the disease pathogenesis.

## 2. Molecular Basis of Trypanosomatid-Host Cell Interaction

Metacyclogenesis is an important process for parasite virulence. This mechanism allows trypanosomatids to infect their vertebrate host and thus their host cells [7]. Inside the vector gut,* Leishmania* parasites transform from procyclic promastigotes to metacyclic promastigotes during metacyclogenesis [7], whereas* T. cruzi* transitions from epimastigotes to metacyclic trypomastigotes [8].

For a long time,* Leishmania* spp. was believed to be obligatory intracellular pathogens of macrophages. However, recent studies have shown that these protozoa infect a large range of host cells [9–11]. Various groups have shown that these parasites can infect multiple cell types* in vitro* as well as* in vivo*, from haematopoietic cells that arise from a common myeloid precursor to nonhaematopoietic cells, such as fibroblasts [10].

Early in infection, neutrophils are recruited in response to a bite from the insect vector due to the release of the alarmins (signal for tissue damage), cytokines, and chemokines [10, 12]. These cells can act against the intracellular microorganisms through reactive oxygen species (ROS) [13, 14], neutrophil elastase (NE), and neutrophil extracellular traps (NETs) [15]. Nevertheless, if these mechanisms can be evaded, neutrophils may serve as host for* Leishmania* parasites. They are infected by promastigotes during the first 18 hours. These cells undergo apoptosis, and the apoptotic bodies are phagocytized by macrophages, triggering anti-inflammatory signal pathways. This results in the silent entry of the parasites inside macrophages, which promotes infection success [16]. It is interesting to note that neutrophils readily phagocytized promastigotes, but recognition or uptake of amastigotes has not been detected yet [17].

The initial binding and internalization of the* Leishmania* promastigotes is a classical receptor-mediated endocytic event that involves serum-derived factors as well as parasites and host cell molecules. The major macrophage plasma membrane structures involved in this interaction are (1) receptors for the complement component 3 subunits C3b and C3bi, which bind to CR1 and CR3, respectively; (2) Fc receptors; (3) lectin receptors, which mediate connections with carbohydrate molecules; and (4) the integrin family of molecules that recognize specific amino acid sequences. The major surface molecules of* Leishmania* that may also participate in this interaction include gp63 or promastigote surface protease (PSP), the primary parasite surface protein; lipophosphoglycan (LPG), the main promastigote glycoconjugate; and glycosyl inositol phospholipids (GPIs), which are present in large numbers in both promastigotes and amastigotes [18].

The parasite surface molecules responsible for the independent binding of serum are LPG, gp63, and glyco inositol phospholipids (GIPLs). In* L. major*, LPG is involved in the invasion of both promastigotes and amastigotes, although this molecule is absent in amastigotes of certain parasite species. Proteophosphoglycan (PPG) is particularly important in the invasion of macrophages by a number of* Leishmania* amastigotes [19, 20].

**Figure 1:**
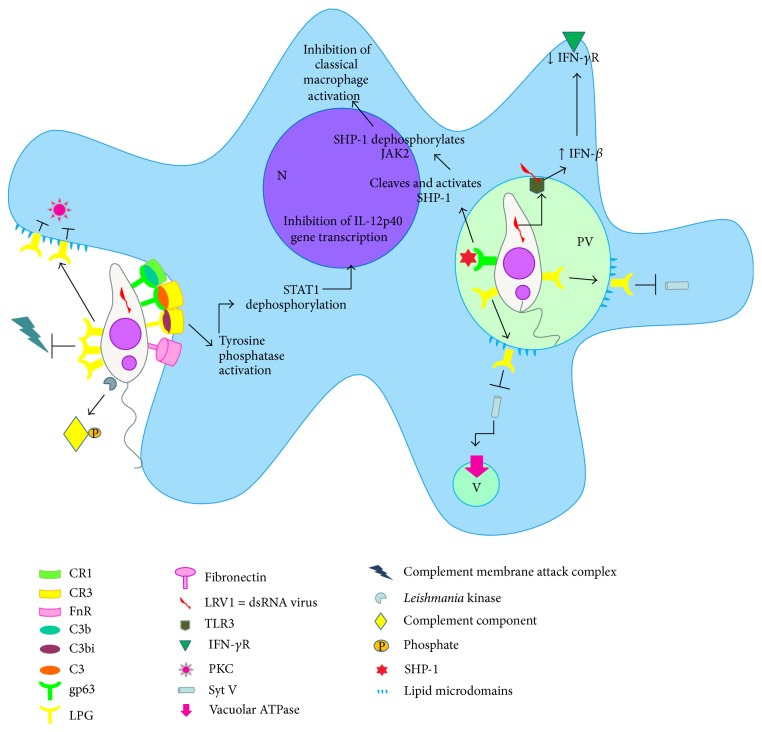
*Leishmania* survival and host cell modulation: The LPG coating of the parasites prevents the complement membrane attack complex insertion. In addition, the promastigote kinase phosphorylates the components of the complement, inhibiting its activation. Promastigote opsonized by C3bi interacts with macrophage membrane CR3 activating tyrosine phosphatase that dephosphorylates STAT-1 leading to inhibition of transcription of the IL-12p40 gene. During the process of promastigote internalization LPG is transferred from parasite membrane to host cell membrane promoting lipid microdomain disruption inhibiting PKC activation and ROS generation (burst oxidative). Inside the parasitophorous vacuole (PV) membrane, this disruption causes the exclusion of synaptotagmins V (Syt V), abrogating the recruitment of the vacuolar ATPase and, consequently, PV acidification allowing the survival of promastigotes. The* Leishmania* dsRNA virus (LRV1) binds toll-like receptor 3 (TLR3) triggering strong IFN-*β* production and downregulation of IFN*γ*-R. Already gp63 cleaves SHP1 prevents classical macrophage activation by IFN-*γ*. N—nucleus, PV—parasitophorous vacuole and V—vacuole.

Both LPG and GIPLs are capable of binding to a mannan-binding serum protein (MBP), which is able to activate the complement system in an antibody-dependent manner. This mechanism may be particularly important in the case of amastigotes that have little or no LPG and gp63 on their surface [18]. On the other hand, gp63 and LPG act as acceptor sites for the complement component 3 (C3) and interact with CR3 and p150, 95, members of the CD18 family of integrins [21, 22]. Meantime, some studies demonstrated that internalization of promastigotes of LPG-defective* Leishmania* is higher than of wild-type (WT) promastigotes [23–26]. Thus, it seems unlikely that LPG plays an essential role in promastigote adhesion to macrophages, but it appears that may interfere with the process of phagocytosis. For accommodating the plasma membrane extension that occurs during the phagocytosis of large particles, as the parasites, focalized exocytosis of endomembrane occurs at the phagocytic cup [27–29]. Several intracellular compartments, including endoplasmic reticulum, late endosomes, and recycling endosomes may contribute to membrane formation of the phagosome through fusion events regulated by soluble N-ethylmaleimide sensitive factor attachment protein receptors (SNAREs), such as VAMP3, VAMP7, and syntaxin 18 [30–35]. The activity of SNARE is regulated by synaptotagmins (Syts), a family of transmembrane proteins that act as sensors of Ca^2+^ [36, 37]. The first Syt protein characterized in phagocytosis is the lysosomal Syt VII, which regulates Ca^2+^-dependent exocytosis of lysosomes [38] and directs the lysosomal membrane to the phagosome [39]. Another protein was posteriorly identified as Syt V, a recycling endosome associated protein recruited to forming phagosome and controls the phagocytic process [40]. After* Leishmania*-host cell contact, LPG is transferred from the parasite to the macrophage membrane during phagocytosis and seems to promote blockage of macrophage activation, protecting the parasite [41]. This insertion promotes disruption of existing lipid microdomains and alters the formation of these structures after promastigote internalization [42, 43], causing the exclusion of Syt V [44]. Consequently, LPG impairs the recruitment of Syt V to the nascent phagosome, resulting in a reduction in the phagocytic capacity of host macrophages [45]. However, the Syt V exclusion from phagosomes promoted by LPG abrogated the recruitment of the vacuolar ATPase and, consequently, their acidification [44], creating a hospitable intracellular niche for* Leishmania *(Figure 1). Thus, although the entry of parasites into macrophages is reduced, their higher survival is reached due to lack of the phagosome acidification and this may represent a larger gain in overall adaptation of these protozoa.

In addition to vector transmission, infection by* T. cruzi* can also occur through organ transplantation [46], blood transfusion [47], congenital transmission [48], oral transmission [49], or laboratory accidents [50]. The literature has suggested that host cell invasion requires the activation of signal transduction pathways that lead to an increase in cytosolic calcium concentration in both the parasite and the host cell and the recruitment and fusion of host perinuclear lysosomes to the site of invasion [51, 52]. According to Andrews [53], the trigger for host cell calcium production is the recruitment of perinuclear lysosomes to the* T. cruzi* invasion site. At this site, lysosomes are incorporated immediately into the parasitophorous vacuole without polymerized actin accumulation, and invasion is facilitated by disruption of microfilaments. However, the recruitment of lysosomes is not currently believed to be essential in this process but is essential for parasite persistence in the host cell. In professional phagocytes, parasite internalization occurs by conventional phagocytosis. Following the adhesion of the parasite to the host cell membrane, molecular signals are triggered, initiating this process. The invasion efficiency in nonphagocytic cells varies among the different developmental forms, that is,* T. cruzi* strains and phylogenetic lineages. Extracellular amastigotes, for example, are potent inducers of phagocytosis in nonprofessional phagocytes, a process that may facilitate parasite persistence in infected hosts [54].

**Figure 2:**
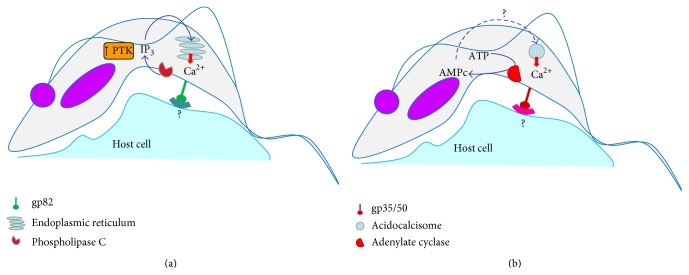
Activation of different signaling pathways for host cell invasion by* T. cruzi* II (a) and* T. cruzi* I (b). (a) The ligation gp82-receptor activates a protein tyrosine kinase and a parasite phospholipase C, which releases Ca^2+^ from inositol-1,4,5-triphosphate (IP3) sensitive reservoirs, possibly the endoplasmic reticulum. (b) The gp35/50-receptor binding appears to stimulate adenylate cyclase activity that seems to participate in Ca^2+^ release from acidocalcisomes.

Trypomastigotes adhere to host cells using surface receptors. Surface glycoproteins such as gp82 and gp35/50, which induce calcium-mediated signaling, are utilized differently among different strains of* T. cruzi*. Isolates that enter the host cell in a gp82-dependent manner (*T. cruzi* II—endemic areas) activate a protein tyrosine kinase and a parasite phospholipase C, which releases Ca^2+^ from inositol-1,4,5-triphosphate (IP3) sensitive reservoirs, possibly the endoplasmic reticulum (Figure 2(a)). However,* T. cruzi* isolates that bind to target cells using gp35/50 (*T. cruzi* I—Amazon region) appear to stimulate adenylate cyclase activity that seems to participate in Ca^2+^ release from acidocalcisomes [55, 56] (Figure 2(b)). Metacyclic trypomastigotes trigger Ca^2+^ release from intracellular stores sensitive to IP3 in the host cell and induce Ca^2+^-dependent disorganization of actin cytoskeleton. The Ca^2+^ release also mobilizes perinuclear lysosomes to the site of* T. cruzi* invasion. Some studies report that lysosomes that fuse directly to the vacuole are already in the plasma membrane. Thus, Ca^2+^ would act only in the fusion and not in the recruitment in this case. Ca^2+^-dependent lysosomal exocytosis is regulated by cAMP and is increased by isoproterenol, a *β*-adrenergic agonist that activates adenylate cyclase. This mechanism appears to be used by the cell to repair cellular membrane (Figure 2) [57]. In the early 2000s, some studies suggested that Syt VII, which is located on the membrane of lysosomes and regulates exocytosis of these organelles, appears to participate in the invasion process of* T. cruzi* [58].

**Figure 3:**
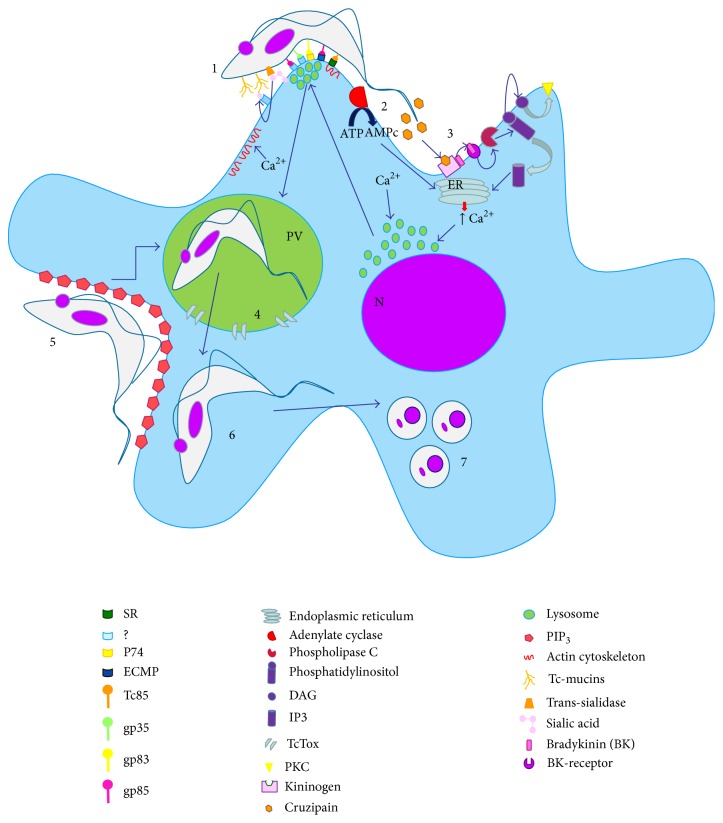
General molecular mechanisms for host cell invasion by* Trypanosoma cruzi*. (1) Several receptor-ligand complexes seem to participate in* T. cruzi* internalization by the host cell. The enzyme transsialidase transfers sialic acid from host cell membrane to Tc-mucins. These molecules can interact with host cell receptor. Some host cell receptors remain unknown. Glycoproteins from the parasite (gp 83 and gp 85, resp.) can bind to host cell receptors, such as P74 or EMCP. Members of Tc85 family can bind to specific receptor (SR) promoting cytoskeletal changes and facilitate parasite invasion. (2) During the process, the host cell adenylate cyclase is activated promoting an enhancement of AMPc that contributes to Ca^2+^ release from endoplasmic reticulum. (3) The cruzipain secreted by the parasite cleaves the host kininogen to liberate bradykinin, and the triggering of the host bradykinin receptor activates host cell PLC, contributing to Ca^2+^ release, via IP_3_. (4) Ca^2+^ seems to promote recruitment of perinuclear lysosomes that contributes to formation of the parasitophorous vacuole. In addition, Ca^2+^ promotes disorganization of actin cytoskeleton, and invasion is facilitated by disruption of microfilaments. (5) In another route, the parasite enters into host cell by creating an invagination in the plasma membrane, which accumulates PIP3. (6) After the PV formation, TcTox promotes pores in their membrane and the trypomastigotes escape for cytoplasm. (7) Trypomastigotes transform into amastigotes, multiplying inside the cytoplasm from host cell. N—nucleus, PV—parasitophorous vacuole.

A new lysosome-independent route of host cell invasion has recently been described. In this route, the parasite enters into host cell by creating an invagination in the plasma membrane, which accumulates phosphatidylinositol-3,4,5-triphosphate (PIP_3_), the main product activation of phosphatidylinositol-3-kinase class I (PI_3_K) (Figure 2) [56]. In a quantitative analysis of the ways in which trypomastigotes of* T. cruzi* penetrate into the host cell, 20 to 25% of trypomastigotes were observed to enter the lysosome exocytosis- and Ca^++^-dependent pathway, approximately 50% invaded via the PI_3_K-dependent pathway and remained in a vacuole formed only by the plasma membrane for an initial period, and approximately 20% entered using the PI_3_K-dependent pathway and quickly associated with primary endosomes [56]. However, independently of the entry mechanism, all parasites are found in parasitophorous vacuole-associated lysosomes within 60 minutes because this fusion is essential for* T. cruzi* survival [59]. Unlike in many other intracellular parasites that avoid fusion with host cell lysosomes [60], this process is a prerequisite for the survival of* T. cruzi* [56]. If the parasite does not associate with these organelles, the persistence of parasites in the host cell is seriously compromised, and the entry process is reversed [56]. The exposure of trypomastigotes to this acidic environment is essential for the activity of the porin-like protein TcTOX; this protein is responsible for parasitophorous vacuole lysis and parasite escape into the cytoplasm, which is necessary for the differentiation of trypomastigotes into amastigotes that begins within the low vacuole pH (Figure 3) [55, 61].

Because* T. cruzi* is unable to synthesize sialic acid, the only mechanism for sialylation of their membrane glycoproteins is to transfer the sugar from host cell glycoconjugates through the action of enzymes. This phenomenon is important for host cell interaction and parasite internalization [62, 63]. Trypomastigotes express large amounts of a protein with transsialidase (TcTS) activity on their surface [64]. The transsialidase is bound to the parasite membrane through a glycosylphosphatidylinositol (GPI) anchor and acts to specifically catalyze the transfer of sialic acid from glycoconjugate proteins from the extracellular environment to mucin-associated surface protein (MASP) that cover the surface of the parasite (Tc-mucins) [65], which is important to promote the parasite entry and persistence in the mammalian host cells (Figure 3). These protein domains are rich in threonine residues [66, 67]. These residues can be modified by protein glycosylation, which is an important posttranslational modification for host-parasite interactions. The* O*-glycosylation of* T. cruzi* mucins (Tc-mucins) is initiated by enzymatic addition of *α*-*O*-N-acetylglucosamine (GlcNAc) to threonine (Thr) by the UDP-GlcNAc: polypeptide *α*-N-acetylglucosaminyltransferase (pp-*α*-GlcNAcT) in the Golgi [68]. These* O*-glycans are the acceptors of sialic acid, as already cited by the literature [69, 70]. The different evolutive forms of* T. cruzi* present different molecular masses of Tc-mucins. Epimastigotes and metacyclic trypomastigotes (MT) present Tc-mucins with molecular mass varying from 35 to 50 kDa, while the Tc-mucins from trypomastigotes derived from cell culture (TCT) the variation range is between 60 and 200 kDa [71, 72]. These masses are compatible with glycosylated protein containing sialic acid, which is essential for host cell binding and invasion [72]. These differences seem to contribute for differential susceptibility of MT and TCT to pepsin digestion. The mucin-like molecules that covered the MT are resistant to proteolysis and protect the parasites from lysis in the gastric milieu [73], in the meantime TCT are susceptible to peptic digestion and are mostly lysed (90%) when incubated with pepsin at pH 3.5 for 30 min [74]. In addition Tc-mucins from TCT are capable to induce NO, IL-12 and TNF-*α* by activated macrophages [75], modulating the immune response during* T. cruzi* infection.

The gene superfamily gp85/trans-sialidase (TS) encodes several glycoproteins that are present on the surface of the parasite and can participate in cell invasion. One of these glycoproteins is called gp83 and plays an important role in the interaction of* T. cruzi *with the host cell interacting with the p74 receptor present on the surface of the host cell and acting as a universal ligand for* T. cruzi *infection of both, phagocytic and non-phagocytic cells [76]. The Tc85 molecules are involved in the adhesion of parasites to the host cell by laminin and other extracellular matrix proteins (ECMP), which can be anchored to the plasma membrane (Figure 3) [77].

A synthetic peptide based on the conserved FLY domain (VTVXNVFLYNR) present in all members of the gp85/TS family promotes dephosphorylation of an intermediate filament protein (cytokeratin 18) that leads to cytoskeletal reorganization facilitating entry of the parasite [78]. This mechanism also promotes activation of the ERK1/2 signaling cascade, resulting in an increase in parasite invasion in epithelial cells [79]. However, an inactive form of TS from TCT that binds sialic acid has been shown to trigger NF-*κ*B activation, the expression of adhesion molecules on endothelial cells and upregulation of parasite entry in an FLY-independent and carbohydrate-dependent manner [80].

The gp90 protein, an N-glycosylated protein [81] with a GPI anchor [82, 83], as well as cruzipain are also involved in host cell invasion of metacyclic trypomastigotes. Secreted cruzipain cleaves host kininogen to liberate bradykinin, and the triggering of the host bradykinin receptor activates host cell PLC, contributing to Ca^2+^ release (Figure 3) [81, 84]. In epimastigotes, cruzipain appears to be linked to degradation processes and localization in the endosomal-lysosomal system [85] but has been described as playing a role in adhesion [86] and cell invasion in trypomastigotes [87].

## 3. Immune Response against* Leishmania* and* Trypanosoma cruzi* and Their Evasion Mechanisms 

The immune system recognizes and responds to a broad spectrum of pathogens, including microorganisms such as viruses, bacteria, fungi, and protozoan parasites, and multicellular parasites, such as helminthes and ectoparasites. Vertebrates possess two types of immunity: innate and adaptive. The innate immune response involves the innate lymphoid cells (ILCs), which are lymphoid cells that do not express rearranged receptors. These cells present essential effector and regulatory functions in innate immunity and tissue remodeling. Two model members of ILC family are natural killer (NK) cells and lymphoid tissue inducer (LTi) cells. The ILCs are divided into 3 groups. This classification is based on their pattern of cytokines produced and the transcription factors required for their development. Group 1 ILCs (ILC1s) produce interferon *γ* and depend on Tbet, group 2 ILCs (ILC2s) produce type 2 cytokines like interleukin-5 (IL-5) and IL-13 and require GATA3, and group 3 ILCs (ILC3s) include lymphoid tissue inducer (LTi) cells, produce IL-17 and/or IL-22, and are dependent on ROR*γ*t [88]. NK cells were classified into ILC1s group because they produce IFN-*γ* [89] and recent information about ILCs development in mouse suggests that NK cells can be considered as the innate form of TCD8 (T cytotoxic) cells as well as CD127+ILCs, the innate form of TCD4 (T helper) cells [90].

The innate response is based on the recognition of pathogen-associated molecular pattern molecules (PAMPs), which are present in diverse organisms but are absent in the host and function as an exogenous signal that alerts the host to the presence of pathogens [91]. The major PAMPs include microbial nucleic acid, lipoproteins, surface glycoproteins, and other membrane components. They are recognized by pattern recognition receptors (PPRs), such as toll-like receptors (TLRs), retinoic acid-inducible gene I- (RIG-1-) like receptors (RLRs), AIM2 like receptors (ALRs), and nucleotide-binding oligomerization domain- (NOD-) like receptors (NLRs) [92]. During infection, PAMPs are recognized by PPRs that initiate signaling cascades that lead to the activation of transcriptions factors in innate immune cells. Macrophages, dendritic cells (DCs), mast cells, and neutrophils are important cells involved in the innate immune response. Innate immune effectors mechanisms include phagocytosis, cytokine and chemokine production, and expression of costimulatory molecules on antigen present cells (APCs) and have an influence on T lymphocyte differentiation [93, 94].

The adaptive immune response involves T and B lymphocytes that recognize a large spectrum of antigens using highly specific receptors. The two major populations of T cells, TCD4 (T helper) and TCD8 (T cytotoxic) cells, have T cell receptors (TCR) that recognize antigens bound to the major histocompatibility complex (MHC) on APC (MHC class II) or target cell (MHC class I) surfaces. Antigen presenting cells such as DCs, macrophages, and B cells express MHC class II molecules and costimulatory molecules on their membranes and present antigen to naive TCD4 cells, whereas MHC class I cells can be also expressed by others cell present antigen to TCD8 cells. After an antigen is recognized, T cells proliferate and differentiate into effector T cell subsets. TCD4 cells orchestrate the immune response by the differentiation into a T helper cell population that secretes distinct sets of cytokines, providing help to B lymphocyte and TCD8 cytotoxic cells. Naive TCD4 cells differentiate into at least four T helper (Th) cell subsets: Th1, Th2, Th17, and regulatory T cells (T_reg_) [95]. DCs play a critical role not only in processing and presenting antigens to naive TCD4 cells but also in secreting cytokines such as IL-12 that induce Th1 effector lymphocyte differentiation. Although DCs are important in the development of Th2 response, other cells such as mast cells, basophils, natural killer cells, and monocytes, secrete cytokines like IL-4 that induce TCD4 differentiation to Th2 [96, 97]. A recent study demonstrates that lung ILC2s enhance effector functions of Th2-type CD4^+^ T cells when they are cultured together* in vitro*. The interaction between ILC2s and CD4^+^ T cells appears bidirectional and likely requires both OX40L and IL-4 and perhaps other molecules. These findings suggest that lung ILC2s and CD4^+^ T cells cooperate to mediate robust Th2-type immune responses in mice [98].

Type 1 responses are characterized by the induction of Th1 cells; these cells secrete cytokines such as interleukin-2 (IL-2) and IFN-*γ*, which are indispensable for host immunity to intracellular parasites (e.g.,* Trypanosoma cruzi*). In contrast, type 2 responses are characterized by Th2 cells that secrete IL-4, IL-5, IL-9, and IL-13 and are induced by and confer immunity to extracellular parasites (e.g., helminths). IFN-*γ* induces cytotoxic TCD8 cell differentiation and macrophage activation, which stimulates the expression of nitric oxide (NO) synthase enzyme (iNOS or NOS2) and the production of NO, the main microbicidal agent able to destroy intracellular parasites such as* Leishmania*. Th2 cells promote B cell responses and immunoglobulin E (IgE) secretion through IL-4 production. In addition to antibody production, B cells have other important functions, such as presenting antigens to T cells and cytokine production. As with T cells, B cells contain functionally distinct subsets with regulatory functions, such as the production of anti-inflammatory IL-10 [99, 100]. The immune system must adjust the magnitude and duration of response because uncontrolled inflammation may lead to immune-mediated tissue injury. Treg cells are important anti-inflammatory cells that are critically involved in limiting the inflammatory response. The suppression of the immune response by Treg cells includes both cell contact- and factor-dependent mechanisms, such as cytokine production (IL-10, TGF-*β*, and IL-35) [101, 102].

The balance between effector and regulatory T cell responses influences the balance between infection control and pathogenesis. Comparing responses exhibited by susceptible and resistant experimental models has contributed to an understanding of protective immune responses to* T. cruzi* and* Leishmania* spp.

After transmission by sand flies,* Leishmania* parasites infect neutrophils, macrophages, and DCs in the vertebrate host, and the development of a protective immune response requires the coordinated action of cells of the innate and adaptive immune response. Generally, protective immunity against leishmaniasis is associated with an inflammatory Th1 response, while disease is associated with an anti-inflammatory Th2 response [103].

Six major* Leishmania* species (*L. tropica*,* L. major*, and* L. donovani*, in the Old World and* L. infantum*,* L. braziliensis*, and* L. mexicana*, in the New World) cause the three main forms of the disease in humans, dermal cutaneous leishmaniasis (CL), visceral leishmaniasis (VL), and muco-cutaneous leishmaniasis or mucosal leishmaniasis (MCL or ML). The form and severity of the disease depend on the* Leishmania* species causing the infection and the immune status of the host [104]. Some of these species have metastatic characteristics and up to 10% of CL cases progress to MCL forming destructive secondary lesions in the mucosa of nose and mouth, in South America. This clinical complication is associated with* Leishmania (Viannia)* subgenus, since it is promoted by species inside of this group, predominantly* L. (Viannia) braziliensis* but also* L. (Viannia) guyanensis* and* L. (Viannia) panamensis* [105]. A common characteristic in the cases of metastatic infection of* Leishmania* is the destructive hyper-inflammatory immune response, caused by numerous activated immune cells, promoting swelling and destroying local tissue [106, 107]. Thus, in ML/MCL exacerbated inflammatory immune response induces tissue injury, and patients present higher levels of proinflammatory cytokines, such as IFN-*γ*, and low levels of anti-inflammatory cytokines, such as IL-10, even after cure compared to the benign cutaneous clinical form of disease [108]. This exacerbated reaction generally is associated to a parasite factor. Although endosymbiont dsRNA virus already have been described on the subgenus* Leishmania (Viannia)* a long time ago [109–111], just recently the presence of this endosymbiont was associated with leishmanial virulence and metastasis [112, 113]. The nucleic acid of* Leishmania* dsRNA virus (LRV1) behaves as a strong innate immunogen, inducing a hyper-inflammatory immune response by pathway of toll-like receptor 3 (TLR3). This pathway induces production of a type 1 IFN response: IFN-*β*-mediated antiviral response. Type I IFNs promote downregulation of IFN*γ*-R on the surface of macrophages. Consequently, the macrophages become insensitive to classical activation, which promotes the reactive nitrogen species (NO) production responsible to kill intracellular pathogens (Figure 1) [114]. In this way, this mechanism is proposed as promoter of the exacerbated MCL phenotype, triggering an increase in the disease severity and parasite persistence [115].

The dsRNA virus seems to interfere with NO production for another via: metastatic* L. braziliensis* species induce higher levels of insulin-like growth factor (IGF) which is able to promote upregulation on the arginase activity [116]. However, this kind of report does not have to investigate the possible influence of LRV infection on oxidative resistance [115].

VL exhibits a mixed type 1 and type 2 cytokine profiles. Studies in experimental models show that neither IL-4 nor IL-13 (typically Th2 cytokines) is able to induce disease exacerbation. However, a consensus exists in relation to the suppressive effect of IL-10 on the immune response in VL and its correlation with disease severity, as this cytokine is an important immunosuppressant and inhibitor of macrophage microbicidal activity in both mice and humans with VL [103, 117]. The control of VL is also dependent on the development of type 1 cytokines and effector antileishmanial molecules such as reactive nitrogen and oxygen intermediates for parasite control in the spleen [118].

In the case of Chagas disease, the existence of a large spectrum of clinical manifestations is associated with parasite heterogeneity and the host immune response. The acute phase is followed by the development of effective acquired immunity, leading to the control of parasitaemia and parasitism levels in tissues. Some authors have shown that in experimental models of acute* T. cruzi* infection, the T helper type 1 response (Th1) appears to have a critical role in infection control. This response can occur through toll-like receptor- (TLR-) mediated and TLR-independent cytokine production [119–121]. Early studies indicated that this infection promotes a certain degree of immunosuppression; however, subsequent data using new approaches demonstrate a substantial antiparasite response during acute experimental infection [122]. Peripheral blood mononuclear cells (PBMCs) from children with acute phase infections present mRNA profiles of interferon (IFN)-*γ*, interleukin (IL)-2, and IL-10, with low levels of IL-4 [123]. Interestingly, children with asymptomatic chronic Chagas disease present upregulated IL-4 mRNA, suggesting that following Th1-mediated parasite clearance, a balance of both Th1 and Th2 immune responses occurs to suppress parasite load and protect against immunopathology [122, 123]. Another study showed that children with acute infection present higher serum levels of tumor necrosis factor (TNF)-*α*, sIL2R, sCD8, sCD4, and IL-6, but no change in IL-2, IL-12, and IL-8 is observed compared to healthy controls or children with asymptomatic Chagas disease in the chronic phase [124].

The lifelong chronic phase is maintained with low parasitaemia and tissue parasitism. The first source of IFN-*γ* seems to be natural killer (NK) cells. This cytokine augments IL-12, TNF-*α* and other cytokines synthesized by classically activated macrophages [125–127]. IFN-*γ* produced by NK cells and IL-12 produced by macrophages have been suggested to induce the differentiation of T-helper cells to a predominant protective Th1 phenotype [128, 129]. IFN-*γ* produced by Th1 cells activates effector mechanisms in macrophages. These effector mechanisms destroy both amastigotes and phagocytized trypomastigotes, whereas cytotoxic activity displayed by CD8^+^ T cells destroys cells containing intracellular amastigotes [130–137].* T. cruzi* antigen-specific CD8 T cells are frequently present in infected mice and humans [137]. Antibodies produced by B cells lyse the extracellular trypomastigote form, facilitate the phagocytosis of parasites opsonized with IgG [138], and promote the complement-dependent killing of the parasites [139]. During severe cardiomyopathy of the chronic phase of Chagas disease, the occurrence of intense inflammatory response is correlated with the production of type 1 cytokines, such as TNF-*α* and IFN-*γ*. In this disease phase, the level of these cytokines is higher than during indeterminate phase of Chagas disease [140]. Indeterminate patients seem to have a more regulated response by Treg cells, limiting tissue damage with the maintenance of improved cardiac function, but apparently the mechanism is not IL-10- or CTLA-4-dependent [141]. These essential responses to Chagas disease have been clearly shown using experimental models or natural human infections in that the absence or the reduction in any of these immune responses (via targeted depletion, immunosuppressive treatments, or infection-induced immunosuppression) can exacerbate parasitaemia [142–144]. In summary, the literature indicates that the persistence of protozoans is related to the delayed kinetics of CD8^+^ T cell development and the balance between Th1 and Th2 responses. An efficient protective response against* T. cruzi* requires the Th1 response, activation of phagocytes, T-helper cells and cytotoxic T lymphocytes, and lytic antibodies.

The ability of parasitic protozoa to interfere with effector mechanisms of the immune response has been studied over decades. The complex life cycle of* Leishmania* sp. and* T. cruzi* involves the emergence of a number of characteristics that allow its survival in different microenvironments in the insect vector and the vertebrate host. Polyclonal lymphocyte activation is an example of an immune evasion mechanism found in some pathogens. In a mouse model of* T. cruzi* infection, reduced levels of polyclonal lymphocyte responses correlate with infection and control of cardiomyopathy. The enzyme proline racemase was described in* T. cruzi* (TcPRAC) and is expressed either as a cytoplasmic, membrane-associated protein [145] or as a secreted isoform, which can be detected at all stages of the parasite life cycle. The secreted form is shown to be a B cell mitogen, which contributes to parasite evasion of the host immune system and its persistence in the vertebrate host [146].

The vertebrate infective forms have developed several strategies to survive in the hostile host environment. For example, bloodstream* T. cruzi* trypomastigotes express molecules on their surface that are capable of interfering with the activation of the classical and alternative complement pathways [147]. Several membrane glycoprotein-specific trypomastigotes participate efficiently and prevent complement activation on the surface of the parasite. Some of these molecules, such as gp160, gp58/68, and T-DAF, regulate complement by inhibiting the development and/or accelerating the decay of C3 convertase, a central enzyme in the complement cascade [148]. Recent studies [149] provide evidence that metacyclic trypomastigotes induce the formation of vesicles derived from the host cell, which form a complex released from the parasite surface, leading to stabilization and inhibition of C3 convertase resulting in increased survival of the parasite.

Another mechanism for the attachment-independent invasion of trypomastigotes involves the activation of the TGF-*β* signaling pathway [150]. A protease secreted by the parasite is likely to activate latent TGF-*β* associated with extracellular matrix (ECM) components, allowing activation of Smad 2/3 pathway through the TGF-*β* receptors (I and II) present on the surface of host cells. The pivotal role of this pathway in infections of heart tissues and, consequently, in the chagasic cardiomyopathy has previously been described [150].

*T. cruzi* has a gene, FL-160, encoding the C-terminus of flagellar protein. This protein has a twelve amino acid epitope similar to nervous tissue proteins present in the sciatic nerve plexus and mesenteric SNC. This gene belongs to a family of highly related genes that are encoded in more than 750 copies in the parasite genome; sequential analyses reveal that all copies of this gene have the 12 amino acids that mimic the human sequence, which may be a prevalence and immunosuppression factor in Chagas disease [151].

In the* T. cruzi* experimental murine acute infection model, several changes are observed in lymphoid organs, including the thymus, where intense and severe thymic atrophy due to depletion of CD4^+^8^+^ double-positive cells (DP) thymocytes by apoptosis in the cortical area of the thymus occurs [152]. A recent study shows that* T. cruzi* trans-sialidase (TcTS) induces thymic atrophy that affects the dynamics of intrathymic thymocytes, resulting in an increase in the number of CD4^+^8^+^ DP recent thymic emigrants in the spleen. TcTS is also capable of activating MAPK JNK signaling in thymocytes, modulating their adhesion to thymic epithelial cells and their migration toward the extracellular matrix. These data suggest the possible involvement of this enzyme in abnormal thymocyte trafficking inside the thymus of animals acutely infected by* T. cruzi*, which could influence the escape of immature thymocytes to peripheral blood in Chagas disease. The authors report that the frequency of DP T cells in chronic patients presenting high antibody titles against TcTS with the cardiac form of Chagas disease is increased. Thus, the presence of peripheral activated DP cells with potentially autoreactive TCRs may contribute to the immunopathological events found in this disease [153].

*T. cruzi* can also interfere with signaling via new members of the B7 family, such as the programmed death ligand 1 (PD-L1). This ligand binds to the programmed death 1 (PD-1) receptor, which is expressed on activated T cells, B cells, and myeloid cells. Their interactions result in downmodulation of the T-cell response [154, 155].* T. cruzi* infection promotes an increase in expression of PD-1 and its ligands on peritoneal macrophages as well as during* in vitro* infection. Macrophages from mice infected by this protozoan are able to promote suppression of T cell proliferation. This suppression is restored when anti-PD-1 and anti-PD-L1 antibodies are added. Additionally, the blockage of PD-1 and PD-L1 increases iNOS expression and NO production on peritoneal macrophages from* T. cruzi*-infected mice [156].

*T. cruzi* infection promotes the formation of lipid bodies in macrophages through TLR2 signaling, which is amplified by the uptake of apoptotic cells in a mechanism dependent on integrins and TGF-*β* synthesis and results in an increased parasite survival and proliferation [157].

For leishmanial infection to be successful, the parasite must resist the hostile environment inside the host and survive to its innate and acquired immune response. Initially these include complement system, followed by phagocytosis, acidification of the phagolysosome, ROS release and, finally, NO production.* Leishmania* promastigotes abundantly express LPG (lipophosphoglycan), which forms a great glycocalyx surround the parasite and interferes with the insertion of membrane attack complex [158]. Promastigotes also present specific kinases able to deactivate the classical and alternative complement pathway by phosphorylating of complement components [159]. In addition gp63 can convert C3b (complement subunit 3), attached to parasite surface, into its inactive form, iC3b, which prevents parasite lysis via the complement system [160]. Furthermore, iC3b can opsonize* Leishmania* and allow entry in the host cell by binding the receptors CR1 and CR3 (complement receptors) (Figure 1) [161, 162].

Inside the neutrophils, the first cell to phagocyte the promastigotes, LPG and an acid phosphatase resistant to tartrate, present on the parasite cell surface, inhibit lysosome fusion and the respiratory burst [163–165]. Promastigotes release a chemotactic substance, lipid* Leishmania* chemotactic factor (LCF) to attract more neutrophils [166]. The LCF can interact with lipoxin A4 receptors (ALX), resulting in the deactivation of oxidative burst of neutrophils [167]. Furthermore,* Leishmania* presents an inhibitor of serine peptidase capable to inhibit the serine peptidase released by neutrophils, the neutrophil elastase. This is crucial for intracellular parasite survival [168].

The Trojan horse theory supports the idea that apoptotic neutrophils infected by* Leishmania*, when taken up by macrophages, allow a silent entrance of the parasite, contributing to the success of infection [169]. Peters et al. [16] showed robust neutrophil infiltration after the bite of the sand fly (vector insect of leishmaniasis) infected with* L. major*. These parasites survive and appear to be better adapted to resist macrophages that were committed during the clearance of apoptotic neutrophils and thus with impaired inflammatory functions when released by neutrophils. Apoptotic* Leishmania* parasites seem to be essential for disease development, because when these cells were depleted from a population of virulent* L. major*, experimental infection was controlled both* in vitro* and* in vivo*. This is due to the increased production of TGF-*β* induced by apoptotic promastigotes [170].

After neutrophils,* Leishmania* promastigotes can enter into dermal macrophages, which lack the respiratory burst machinery. Thus, promastigotes have opportunity to ameliorate their ability to transform into amastigotes and grow inside the host cell [171]. In the same way, promastigotes and amastigotes can be actively ingested by the skin fibroblasts [172], where they find a safe environment for up to 7 days after infection, since these cells produce low levels of NO even in the presence of interferon-*γ*, compared to macrophages [172].

Inside the macrophage, LPG can inhibit the oxidative burst initiated by NADPH oxidase at the time of their entry into the host cell. This phenomenon seems to be another strategy used by* Leishmania* to buy time to transform into the resistant amastigote form. LPG can inhibit PKC (protein kinase C), a key enzyme for initiation of the oxidative burst. LPG is known to inhibit the translocation of this enzyme to the cell membrane due to its transference from parasites membrane to the macrophage membrane during phagocytosis and binds to the regulatory domain of PKC, inactivating the production of reactive oxygen species (ROS) (Figure 1) [173–175]. Furthermore, LPG can inhibit the production of IL-12 by macrophages, thus impairing Th1 differentiation, though the mechanism has not been elucidated [175].

The metalloprotease gp63 is also involved in parasite protection within the phagolysosome. This protease activity appears to protect against host proteolytic enzymes [176]. This important protease is also involved in processes of immune evasion. Gp63 cleaves and activates the protein tyrosine phosphatase SHP-1, interfering with the IFN-*γ* signaling pathway by dephosphorylating Janus kinase 2 (JAK2) (Figure 1) [177]. This JAK2 dephosphorylating negatively interferes with ERK1/2 (extracellular signal-regulated kinase 1/2), MAPK (mitogen-activated protein kinase), nuclear factor-*κ*B (NF*κ*B), IRF-1 (interferon regulatory factor-1), and AP-1 (activator protein 1), inhibiting classical macrophage activation and impairing the production of IL-12, NO, and immunoproteasome formation [178, 179]. NF*κ*B is a key transcription factor that mediates innate and adaptive immunity and is involved in the transcription of adhesion molecules and chemokines that leads to the recruitment and activation of effector cells. GP63 in* Leishmania* promastigotes can cleave the p65^RelA^ subunit of this transcription factor, resulting in the p35^RelA^ fragment that is associated with promotion of certain chemokines (MIP-1*β* and MIP-2—macrophage inflammatory protein) and favors the recruitment of phagocytic cells but does not induce other macrophage products such as iNOS and IL-12, essential for a protective response [180, 181].* Leishmania* may also stimulate the degradation of STAT-1 (signal transducer and activator of transcription) in host cells by modulating signaling pathways through receptors CR3 and FcуR, which inhibits iNOS expression and NO production, leading to parasite survival [182, 183].

In summary,* Leishmania* parasites have a very complex repertoire of strategies to escape the immune system by interfering in a range of signal transduction pathways in host cells (mainly macrophages), inhibiting the protective response and continuing the life cycle.

## 4. Therapeutic Targets

There are large differences between the trypanosomatids and mammalian cells, so different biochemical pathways of parasites from the hosts would be excellent targets for the new drugs design [184]. With the post genomic era, the discovery of new targets can be amplified and supporting the development of drugs more specific for the parasite and less toxic for the host. Among these possible targets include (a) mitochondrial markers; (b) fatty acids, sterols, carbohydrates, and folate biosynthesis [185, 186]; (c) recovery and metabolism of purines, pyrimidines [187], and aminoacids (as proline) [145]; (d) biosynthesis, transport, and metabolism of polyamines [188]; (e) the cell cycle [189, 190]; (f) proteases [191] and (g) proteasomes [192, 193].

Current therapeutic strategies in leishmaniasis and trypanosomiasis are far from satisfactory. The efficacy is variable, toxicity is high, and the emergence of resistance is increasingly common.

The trypanosomiasis treatment dates back over 50 years and is based on nifurtimox and benznidazole, which belong to the class of nitroaromatic compounds. These agents function as pro-drugs and must depend of enzyme-mediated activation inside parasites. The nitroreductases mediate reduction of the nitro-group generating an unstable nitroradical that, in presence of oxygen, generate superoxide. The* T. cruzi* sensibility depends on its capacity detoxification of free radicals as well as associated to downregulation of type I nitroreductases of the parasites [194]. Although benznidazole is considered to be better tolerated than nifurtimox, various adverse effects are attributed to their use, such as neuropathy and agranulocytosis. These drugs are active against blood forms of* T. cruzi* and effective in treating the acute phase of infection; however their efficiency in chronic phase is controversial. Actually, studies are being done to make nitro drugs selectively toxic to the parasite and more effective in chronic phase [195]. Fexinidazole is a nitroheterocyclic effective oral treatment of acute and chronic experimental* T. cruzi* infection [196]. Recently a study showed the efficacy of the metabolites fexinidazole in a mouse model of acute infection, leading to reduced inflammation in heart tissue associated with the chronic phase of Chagas disease [197].

In general, the first line leishmaniasis treatments are pentavalent antimonial that have been developed over 50 years ago. To exert its antileishmanial activity, the pentavalent antimony needs to be reduced to its trivalent form inside macrophages. The mechanism of action of antimonials is not completely clarified, but is known to involve inhibition of glycolitic pathway, fatty acids and trypanotione reductase [198, 199]. Pentamidine, amphotericin B, miltefosine, and paramomycin are used as second-line drugs [200–203]. The action mechanism of pentamidine is not well characterized, although there is evidence that involves mitochondrial functions interference [204]. The paromomycin mechanism is based on inhibition of protein synthesis of the parasite [203], covalently bound to protein and effects translation and vesicle-mediated trafficking [205]. The miltefosine acts on the cell signal transduction pathway by inhibiting protein kinase B, which makes an important role in the biosynthesis of sterols and phospholipids [206]. Already amphotericin B binds to ergosterol, a major component of the cellular membrane of* Leishmania*, forming transmembrane channels that release monovalent ions (K^+^, Na^+^, H^+^ and Cl^−^) leading to cell death [207]. To minimize the adverse events of amphotericin B, various lipid formulations have been introduced leading rapidly concentrated into organs such as liver, spleen and increase the antileishmanial activity with selectivity to macrophage reticular-endothelial system [208]. Sitamaquine (8-aminoquinoline) is oral drug for the treatment of visceral leishmaniasis which has completed Phase II trials in India and Kenya [208, 209]. The molecular targets of sitamaquine are still unknown, however it was shown that upon binding to transiently membrane sterols is found in the cytoplasm and induces changes mitochondrial membrane potential [210].

The sterol biosynthesis is a potential drugs target in trypanosomatids since there are some differences between parasite and host. Parasite is entirely dependent of endogenously sterols for survival and growth and cannot use the supply of the host cholesterol. The major product sterol biosynthesis of trypanosomatids is ergosterol and other 24-methyl sterols and the 14*α*-demethylase (CYP51) is key of pathway inhibited by azoles. These azoles have antiparasite action same the antifungal because block the biosynthesis accumulating toxic methylated precursors [211, 212]. Besides the main targets are membranes of mitochondria, the protozoan cell body and flagellum, other important changes take place in the organization of the kinetoplast DNA network and on the protozoan cell cycle leading to cell death [213]. Azoles as ketoconazole and fluconazole demonstrate the efficacy to treat some clinical forms of leishmaniasis [214–216], while posaconazole and ravuconazole have been reported on clinical trials (phase I or II) on* T. cruzi* infection [217–219].

The TcPRAC is a promising target for the development of a new therapy against Chagas disease since parasites are no longer viable when PRAC genes are knocked down or more virulent if PRAC genes are over expressed [220]. Two compounds, which are irreversible competitive inhibitors of TcPRAC, were able to inhibit the mammalian host cell infection [221].

Recently, protease inhibitors used in antiretroviral therapy regimen of high efficiency (HAART) to treat HIV-infected patients have been used to treat trypanosomiasis and leishmaniasis. Studies show that the strategy had good results against* T. cruzi* and different species of* Leishmania* genus [222]. It is believed that these inhibitors affect the proteasomes of parasites responsible for the proliferation, differentiation and intracellular survival of microorganisms [223].

Aspartic peptidase inhibitors used in the current chemotherapy against HIV were able to inhibit the aspartic peptidase activity produced by different species of* Leishmania* spp and induced an increase in the level of reactive oxygen species, triggering parasite death pathways such as programed cell death (apoptosis) and uncontrolled autophagy [224, 225].

Finally, the possibility of different therapies combination against trypanosomatids can be result ameliorates to the low efficiency, high toxicity and especially reduce possibility of parasite resistance [226]. Thus emerged over the last decade this strategy has been tested successfully against others parasites included malaria and tuberculosis agents [227]. The multitarget compounds use against trypanosomatids is another way to reduce resistance to treatment, since drugs with a single target are susceptible to high level of resistance, a result of the mutation of the target protein [197, 228, 229]. Besides the combination chemotherapy, another important therapeutic approach is immunotherapy. The immunotherapy includes the use of biological substances or molecules to modulate the immune responses for the purpose of achieving a prophylactic and/or therapeutic success. The immunotherapeutic agents can exert their effect by directly or indirectly augmenting the host natural defenses, restoring the impaired effectors functions or decreasing host excessive response. The combination of immunotherapy with chemotherapeutic drugs (immunochemotherapy) is showing promise in the treatment of visceral and mucosal leishmaniasis [230–233].

## 5. Concluding Remarks

The complexity of the relationship between intracellular* Leishmania* spp. and* T. cruzi* and their human hosts is a limitation to vaccine and drug design. Heterogeneity within the same parasite species and our limited experimental models make it even more challenging to understand this complex association. However, knowledge is accumulating regarding the molecular machinery of these parasites and the host immune response can be used to change our paradigms and develop new strategies for the treatment and control of these diseases. Treatments that target different points in parasite metabolism are an important strategy to improve efficacy and prevent resistance. In this sense, a combination of drugs for treatment should be encouraged. The use of immunomodulators may also be relevant to restore homeostasis of the host and attenuate tissue damage contributing to the therapeutic success.
